# Effect of a workshop for questionnaire-based surveys on research awareness and motivation among community and hospital pharmacists in Mie Prefecture

**DOI:** 10.1186/s40780-025-00460-3

**Published:** 2025-07-01

**Authors:** Yuki Asai, Yasushi Takai, Toshiki Murasaka, Tomohiro Miyake, Tomohisa Nakamura, Yoshihiko Morikawa, Yuji Nakagawa, Tatsuya Kanayama, Hiroaki Matsuda, Naoki Masuda, Yoshihiro Miki, Takuya Iwamoto

**Affiliations:** 1 Department of Pharmacy, Mie University Hospital, Faculty of Medicine, Mie University, 2-174, Edobashi, Tsu, Mie 514-8507 Japan; 2 Department of Pharmacy, Mie Heart Center Hospital, 222-1, Ooyodo Meiwa, Taki, Mie 515-0302 Japan; 3 Konan Pharmacy, 1874-4 Karasu, Tsu, Mie 514-0315 Japan; 4 Department of Pharmacy, Ise Red Cross Hospital, 1-471-2, Funae, Ise, Mie 516-8512 Japan; 5 Pharmacy, Mie Prefectural Mental Medical Center, 1-12-1, Shiroyama, Tsu, Mie 514-0818 Japan; 6 Ichishi Dispensing Pharmacy Takano Store, 226-7, Takano, Ichishi, Tsu, Mie 515-2504 Japan; 7 Sanai Pharmacy Ikuwa Store, 826-1, Daimon, Ikuwa, Yokkaichi, Mie 512- 0911 Japan; 8 Mie Pharmaceutical Association, 311 Shimazaki, Tsu, Mie 514-0002 Japan

**Keywords:** Questionnaire-based survey, Community pharmacist, Hospital pharmacist, Workshop

## Abstract

**Background:**

Although questionnaire-based surveys enable pharmacists to systematically assess patient needs, healthcare practices, and medication outcomes, it is essential to minimize various biases to ensure that the data obtained from surveys are both reliable and valid. The present study aimed to elucidate whether a workshop on questionnaire-based surveys could enhance research awareness and motivation among community and hospital pharmacists in Mie prefecture.

**Methods:**

The workshop comprised three parts: lecture (15 min), group work (90 min), and presentation (60 min). The participants’ awareness and motivation for questionnaire-based survey was assessed through a questionnaire before and after the workshop, focusing on three questions. A customer satisfaction analysis was also conducted to identify areas for improvement in workshops on questionnaire-based surveys for future workshops.

**Results:**

Response rate of the questionnaire was 100% (16/16 participants). In the question 1 “I think that it would be beneficial to conduct a questionnaire-based survey on daily tasks and present the findings at conferences or publish them in academic journals”, no respondents answered “Disagree” when asked after workshop. In the question 2 “I would like to conduct a questionnaire-based survey if there is a specific theme”, the proportions of respondents selecting “Neutral” (*p* = 0.027) and “Somewhat disagree” (*p* = 0.001) also decreased after workshop, and all participants responded with either “Agree” or “Somewhat agree.” In the question 3 “I think that I can independently design a research project about questionnaire-based survey.”, the proportion of respondents who selected “Agree” significantly increased from 6.3% before-workshop to 25% after-workshop (*p* = 0.003). The customer satisfaction graph revealed that only “Understanding of the lecture” was located in the priority maintenance area. On the other hand, “Time allocation of the presentation” and “Usefulness of the mentor” were located in the priority improvement area.

**Conclusion:**

The present study revealed that a workshop for questionnaire-based surveys enhanced research awareness and motivation among community and hospital pharmacists. Increasing the time allocated for discussions among participants was suggested to enhance participant satisfaction and potentially influence their understanding and skills in questionnaire-based surveys.

**Supplementary Information:**

The online version contains supplementary material available at 10.1186/s40780-025-00460-3.

## Background

Clinical pharmacists play a crucial role in health care by providing evidence-based pharmaceutical care [1]. Enhancing pharmaceutical care requires clinical research to evaluate and improve the daily practices of pharmacists. Our previous survey confirmed the importance of appropriately evaluating daily practices as clinical research is recognized equally across communities and hospital pharmacists in Mie Prefecture [2]. Because efforts by pharmacists to engage in research activities remain insufficient, not only in Mie Prefecture [3] but also across Japan [4], the development of an educational framework to promote research activities focused on the evaluation of daily practices of pharmacists is an urgent priority.

Questionnaire-based surveys support pharmacists in systematically evaluating patient needs, healthcare practices, and medication outcomes [5]. This type of research is particularly valuable for improving patient care and addressing gaps in clinical settings, thereby fostering a culture of continuous improvement in healthcare services. However, it is essential to minimize various biases such as leading and ambiguity biases during the questionnaire development stage [6]. Failure to address these issues may compromise the reliability and validity of the survey results. Therefore, “The Research Activity Promotion Team” in the Mie Pharmaceutical Association planned a workshop to conduct questionnaire-based surveys. Developing high-quality questionnaires may enable accurate evaluation of pharmacists’ daily practices and enhance motivation for research; however, evidence supporting this remains limited.

The aim of this study was to determine whether a workshop on questionnaire-based surveys could enhance research awareness and motivation among community and hospital pharmacists.

## Methods

### Study design and participants

We conducted a prospective cohort study of 16 community and hospital pharmacists who attended the workshop. The workshop information was distributed via e-email to all pharmacies and hospitals belonging to the Mie Pharmaceutical Association in the period from November 5, 2024 to November 20, 2024.

### Details of workshop

The workshop was conducted on December 1, 2024. The timetable is shown in Supplementary Table 1. For the first 15 min, the lecture focused on the design of a pharmaceutical questionnaire. Second, the theme of group work was introduced. Third, the research topics were assigned to three groups (five to six members in each group, with a mentor) with the task of taking 90 min to construct a questionnaire relevant to the topic. (Supplementary Fig. 1). Lastly, a presentation session was conducted to discuss the pharmaceutical questionnaires for 60 min.

### Questionnaire for evaluating the effectiveness of workshop

Questionnaires were collected using Google Forms (Google, Mountain View, CA, USA). Details of the questions are provided in Table 1. The outcome was set as the effectiveness of the present workshop on an increase in the participants’ awareness and motivation of questionnaire-based survey and was evaluated based on three questions. For questions on outcomes, the same responses were obtained before and after the workshop. While questionnaires before the workshop were collected at the time of registration, a second questionnaire survey was performed immediately after the workshop.

**Table 1:** Questionnaire contents for usefulness of workshop on questionnaire-based survey

	Contents
Basic information	
1	Sex
	□ Male, □ Female
2	Age, years old
	□ 20 to 29, □ 30 to 39, □ 40 to 49, □ 50 to 59, □ 60 to 69, □ ≥ 70
3	Pharmacist experience, years
	□ < 1, □ 2 to 5, □ 6 to 10, □ 11 to 20, □ 21 to 30, □ ≥ 31
4	Workplace distribution
	□ Community pharmacy, □ General Hospital / Clinic, □ Others
5	Do you have a specific theme for a questionnaire-based study?
	□ Yes, □ No, □ Unknown
6	How many academic societies (except for the Japan Pharmaceutical Association and the Japanese Society of Hospital Pharmacists) do you belong to?
	□ 0, □ 1, □ 2, □ 3, □ 4, □ ≥ 5
7	Have you ever attended an academic conference, including only audience? How frequently do you attend?
	□ At least once a year, □ Once every few years, □ Once or less
8	Do you have any certifications related to pharmacy practice?
	□ Yes, □ No
9	Do you think joint workshop between hospital and community pharmacists is useful?
	□ Yes, □ No, □ Unknown
10	Have you ever conducted a survey research study after obtaining approval for your research protocol from an ethics review board?
	□ I have conducted one theme as the principal investigator. □ I have participated as a co-investigator but not as the principal investigator. □ I have never conducted one. □ I have not obtained approval but have conducted or assisted with one as part of my professional duties.
11	For those who answered “I have conducted one as the principal investigator” or “I have participated as a co-investigator but not as the principal investigator” in Question 10: In what form did you disseminate the research findings of the survey study to society?
	□ Reported as an academic paper □ Presented at an academic conference □ Neither reported as an academic paper nor presented at an academic conference □ Unknown
Question items (outcome)	
1	I think that it would be beneficial to conduct a questionnaire-based survey on daily tasks and present the findings at conferences or publish them in academic journals.
	□ Agree, □ Somewhat agree, □ Neutral, □ Somewhat disagree, □ Disagree
2	I would like to conduct a questionnaire-based survey if there is a specific theme.
	□ Agree, □ Somewhat agree, □ Neutral, □ Somewhat disagree, □ Disagree
3	I think that I can independently design a research project about questionnaire-based survey.
	□ Agree, □ Somewhat agree, □ Neutral, □ Somewhat disagree, □ Disagree

### Customer satisfaction analysis

A customer satisfaction (CS) analysis was conducted to identify areas for improvement for the design of future workshops on questionnaire-based surveys in pharmaceutical practice. Details of the questionnaire used for the CS analysis are provided in Supplementary Table 2. Based on the CS analysis, Quadrant I was defined as items to be maintained in their current status, whereas Quadrant IV was identified as an item requiring improvement in future workshops. The CS analysis was performed using Microsoft Excel 2019 (Microsoft, Redmond, WA, USA).

### Statistical analysis

Changes in the participants’ awareness of the design and use of questionnaire-based surveys in pharmaceutical practice were analyzed using McNemar’s test. All statistical analyses were performed using SPSS Statistics version 27 (IBM Japan, Tokyo, Japan), and the significance level was set at *p* < 0.05.

## Results

### Background characteristics of respondents

The respondents’ background characteristics are presented in Table 2. The questionnaire response rate was 100% (16/16 participants). Among the participating pharmacists, 87.5% (14/16) were under 40 years of age, with a particularly high proportion having less than five years of experience as a pharmacist (56.3%, 9/16). Half the participants (8/16) had indicated a particular research theme related to questionnaire-based surveys. 75% (12/16) of the participants had never conducted a questionnaire-based survey as principal investigators.

**Table 2:** Background characteristics of respondents

Factor		Respondents, *n*	Rate, %^a^
All (*n* = 16)			
Sex	Male	10	62.5
	Female	6	37.5
Age, years old	20 to 29	8	50.0
	30 to 39	6	37.5
	40 to 49	2	12.5
Pharmacist experience, years	< 1	4	25.0
	2 to 5	5	31.3
	6 to 10	2	12.5
	11 to 20	4	25
	21 to 30	1	6.3
Workplace distribution	General Hospital / Clinic	12	75.0
	Community pharmacy	4	25.0
Have a theme for questionnaire-based survey	Yes	8	50.0
	No	5	31.3
	Unknown	3	18.3
Number of belonging academic conferences	0	5	31.3
	1	1	6.3
	2	6	37.5
	3	2	12.5
	4	1	6.3
	≥ 5	1	6.3
Possession of any certifications	Yes	10	62.5
	No	6	37.5
Frequency of participation in academic conferences	Once or less	7	43.8
	Once every few years	2	12.5
	At least once a year	7	43.8
Usefulness of joint workshops	Yes	15	93.8
	Unknown	1	6.3
Experience in conducting questionnaire-based survey	Principal investigator	4	25.0
	Co-investigator	1	6.3
	Never	11	68.8
Experience in reporting the outcomes of questionnaire-based survey	Academic paper	1	-
	Academic conference	3	-

^a^ (Response / all respondents) ×100

### Effectiveness of workshop

**Fig. 1:**
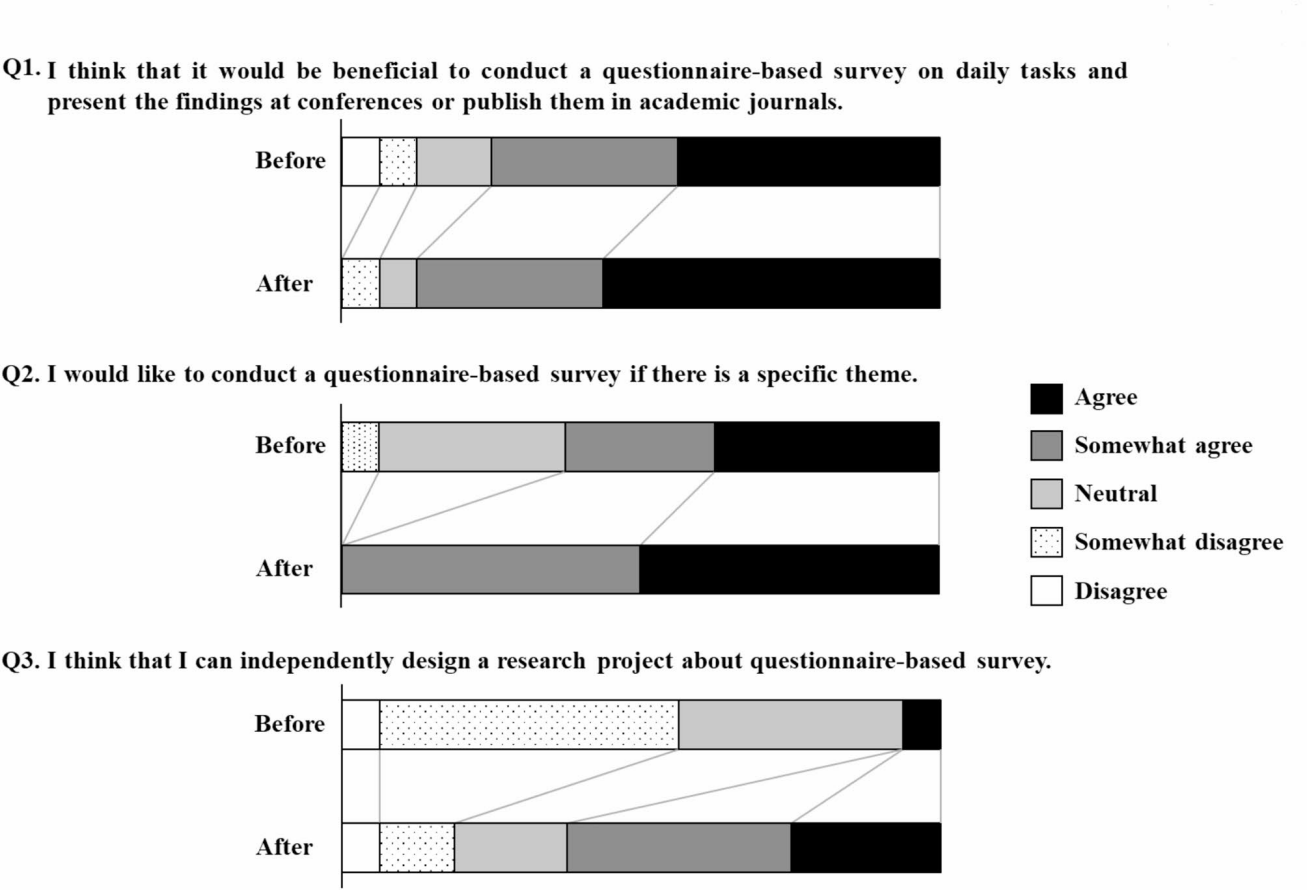
Changes in research awareness and motivation after a workshop on Questionnaire-Based Surveys

The percentage of responses in the post-workshop questionnaire changed significantly from the responses in the pre-workshop questionnaire (Fig. 1). In Question 1, no respondents answered “Disagree” after the workshop. Regarding the willingness to conduct a questionnaire-based survey (Question 2), the proportions of respondents selecting “Neutral” (*p* = 0.027) and “Somewhat disagree” (*p* = 0.001) also decreased, and all participants responded with either “Agree” or “Somewhat agree.” In Question 3, the proportion of respondents who selected “Agree” significantly increased from 6.3% before-workshop to 25% after-workshop (*p* = 0.003), and those who selected “Somewhat agree” significantly rose from 25 to 37.5% (*p* = 0.002).

### Customer satisfaction analysis

**Fig. 2:**
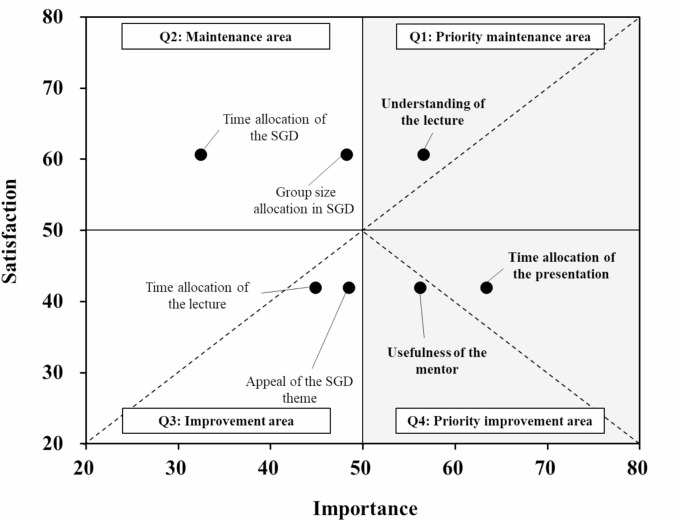
Customer satisfaction after a workshop on Questionnaire-Based Surveys. Q, Quadrant

Regarding satisfaction, 100% were satisfied overall, and more than 94% reported satisfaction with all other items. In the area of priority maintenance (Quadrant 1), “Understanding of the lecture” had only existed (Fig. 2). On the other hand, “Time allocation of the presentation” and “Usefulness of the mentor” were categorized as the priority improvement area (Quadrant 4).

## Discussion

As most participants were early career pharmacists with less than five years of experience and had a low rate of conference participation (Table 2), it was considered that the workshop primarily consisted of pharmacists with limited experience in clinical research. In Japan, the Yakugaku Kyoiku Model Core Curriculum emphasizes research-related skills, particularly ‘Inquiry into pharmaceutical issues and commitment to pharmaceutical research’ and ‘Practice of research’ [7]. Although this highlights the importance of structured training programs, such as the present workshop, the sample size was too small to assess the usefulness of the workshop between early career pharmacists and others, and this remains an issue for future research.

The decrease in negative opinions regarding questionnaire-based surveys and the increase in positive responses (Fig. 1) suggest that the workshop may have contributed to increasing awareness of and motivation toward questionnaire-based surveys in pharmaceutical practice. In this workshop, as 50% of the participants had indicated a specific theme or aspect related to questionnaire-based surveys (Table 2), it may be reasonable to assume that these respondents were already inclined toward a positive opinion about pharmaceutical surveys before attending the workshop. Moreover, all the participants expressed a positive opinion in response to Question 2 after the workshop, suggesting that participation in the workshop was also motivating for those who did not already have a specific research theme in mind.

The CS graph revealed that “Understanding of the lecture” was a high-importance and high-satisfaction item (Fig. 2), indicating that it was considered necessary to continue the content of this lecture. The usefulness of active face-to-face workshops has been reported in clinical research learning methods [8]. In our previous study design workshop in Mie Prefecture, the participants requested more discussion time [2]. Taking these results into consideration, the lecture duration was shortened to 15 min, and the group work session was extended to 90 min. On the other hand, “Time allocation of the presentation” was identified as a high-importance but low-satisfaction item (Fig. 2). This suggests that participants may have desired more time for questions and discussions with those working on other research themes. Mentors’ involvement in theme development may have influenced guidance quality; standardizing mentor instruction was identified as a point for future improvement.

The present study had several limitations. First, because the number of participants in this workshop was limited to 16, it is difficult to generalize the usefulness of the workshop. Second, as the pharmacists who attended this workshop might have already had a high awareness of research activities, the usefulness of this workshop may have been overestimated. Third, because the follow-up questionnaire was administered immediately after the workshop, its results may have yielded higher ratings than if the respondents had had more time to reflect.

## Conclusions

The present study revealed that a workshop for questionnaire-based surveys enhances research awareness and motivation among community and hospital pharmacists. Increasing the time allocated for discussions among participants was suggested to enhance participant satisfaction and potentially influence their understanding and skills in questionnaire-based surveys.

## Electronic supplementary material

Below is the link to the electronic supplementary material.

**Supplementary Material 1**: **Supplementary Table 1**: Contents of the workshop on Questionnaire-Based Surveys. **Supplementary Fig. 1**: Worksheet for group work in the present workshop.

## Data Availability

No datasets were generated or analysed during the current study.

## Abbreviations

CS: Customer satisfaction
