# Supplementary material for: Effect of a workshop for questionnaire-based surveys on research awareness and motivation among community and hospital pharmacists in Mie Prefecture
Source: J Pharm Health Care Sci. 2025 Jul 1;11:55. doi: 10.1186/s40780-025-00460-3 (PMC12219599; doi:10.1186/s40780-025-00460-3)
Supplement: Supplementary file 1 — Supplementary Material 1: Supplementary Table 1: Contents of the workshop on Questionnaire-Based Surveys. Supplementary Fig. 1: Worksheet for group work in the present workshop. [file 40780_2025_460_MOESM1_ESM.docx]

**Supplementary Table 1 Contents of the workshop on questionnaire-based survey.**

| Time schedule (A.M.) | Styles | Details |
| --- | --- | --- |
| 9:00-9:15 | Lecture on the design of questionnaire-based survey | 1) Importance of conducting questionnaire-based survey by pharmacists  2) Key points for designing questionnaires  3) Pitfalls for designing questionnaires |
| 9:15-9:30 | Introduction of the theme for group work | Explanation of the significance and purpose of the group work theme by mentors |
| 9:30-11:00 | Group work (6 persons per theme) | Formulate the clinical problem (target patients, exposure/intervention methods, etc.)  Theme 1: Pharmacists' literacy regarding the OTC analgesics  Theme 2: A survey on patients’ satisfaction with community pharmacies  Theme 3: Pharmacists' literacy regarding dysphagia and swallowing function |
| 11:00-12:00 | Presentation (20 minutes per theme) | One group presentation each study project |

OTC; over the counter

**Supplementary Table 2 Questionnaire contents for customer satisfaction analysis.**

| Contents in CS graph | Questions | Very Low | Low | Neutral | High | Very High |
| --- | --- | --- | --- | --- | --- | --- |
| Understanding of the lecture | Was the lecture helpful for conducting questionnaire-based survey? |  |  |  |  |  |
| Time allocation of the lecture | Was the time allocation of lecture appropriate? |  |  |  |  |  |
| Appeal of the SGD theme | Was the theme of the SGD interesting to you? |  |  |  |  |  |
| Time allocation of the SGD | Was the time allocation of SGD appropriate? |  |  |  |  |  |
| Group size allocation in SGD | Was the group size for the SGD appropriate? |  |  |  |  |  |
| Usefulness of the mentor | Was the mentor important in facilitating smooth discussions during the SGD? |  |  |  |  |  |
| Time allocation of the presentation | Was the time allocation of presentation appropriate? |  |  |  |  |  |
| Over all | Were you satisfied with the workshop on questionnaire-based survey? |  |  |  |  |  |
| If you have any requests or suggestions regarding the workshop, please let us know.  Open-ended response section: (　　　　　　　　　　　　　 ) | | | | | | |

SGD; small group discussion. In the 5-point Likert scale, the top two responses (Very High and High) were defined as “satisfied,” while the bottom two (Very Low and Low) were defined as “dissatisfied.”

**Supplementary Fig. 1**

Group work：Construct the questionnaires

「 Group theme 」

Background：

Purpose for the present theme：

**Tips of constructing the questionnaires**

- **Clearly define the research objective.**
- **Specify the target population.**
- **Ensure that questions are clear and avoid redundancy or ambiguity.**
- **Keep the number of questions to a minimum.**

1.　Let's discuss the target population for the survey!

2.　Clearly define the objectives of the survey. Furthermore, establish their priorities.

3.　Setting the basic information for the survey (respondent demographics).

4.　 Let's design the survey questions!
